# The structural switch of nucleotide-free kinesin

**DOI:** 10.1038/srep42558

**Published:** 2017-02-14

**Authors:** Luyan Cao, Soraya Cantos-Fernandes, Benoît Gigant

**Affiliations:** 1 Institute for Integrative Biology of the Cell (I2BC), CEA, CNRS, Université Paris-Sud, Université Paris-Saclay, 91198 Gif-sur-Yvette, France

## Abstract

Kinesin-1 is an ATP-dependent motor protein that moves towards microtubules (+)-ends. Whereas structures of isolated ADP-kinesin and of complexes with tubulin of apo-kinesin and of ATP-like-kinesin are available, structural data on apo-kinesin-1 in the absence of tubulin are still missing, leaving the role of nucleotide release in the structural cycle unsettled. Here, we identified mutations in the kinesin nucleotide-binding P-loop motif that interfere with ADP binding. These mutations destabilize the P-loop (T87A mutant) or magnesium binding (T92V), highlighting a dual mechanism for nucleotide release. The structures of these mutants in their apo form are either isomorphous to ADP-kinesin-1 or to tubulin-bound apo-kinesin-1. Remarkably, both structures are also obtained from the nucleotide-depleted wild-type protein. Our results lead to a model in which, when detached from microtubules, apo-kinesin possibly occupies the two conformations we characterized, whereas, upon microtubule binding, ADP-kinesin converts to the tubulin-bound apo-kinesin conformation and releases ADP. This conformation is primed to bind ATP and, therefore, to run through the natural nucleotide cycle of kinesin-1.

Kinesins are a family of microtubule-based motors that play important roles in intracellular transport and cell division. Kinesin-1 transports cargo within cells, a process tightly coupled with ATP hydrolysis^1^,^2^. Single-molecule studies have shown that dimeric kinesin-1 moves in a hand-over-hand manner by alternately translocating its two motor domains^3^,^4^. Whereas kinesin-1 in solution is mostly loaded with ADP, ADP release is accelerated several thousand-fold upon microtubule binding^5^,^6^. ATP binding then triggers a conformational change in the microtubule-bound leading motor domain, following which the rear head is drawn forward in the direction of the (+)-end of the microtubule. The moving head then binds to the microtubule 16 nm ahead from its previous position, whereas the (now) rear head hydrolyzes ATP and eventually detaches from microtubule, achieving a step^7^,^8^,^9^.

X-ray crystallographic studies have defined the structures of an ADP-loaded kinesin-1 motor domain^10^,^11^. Structural changes in the nucleotide-binding site upon binding of a non-hydrolysable ATP analog were then identified in the kinesin-5 Eg5 (ref. 12). Most recent X-ray structural studies have shown that a kinesin-1 motor domain comprises three subdomains that reorient as a function of the nucleotide content and upon binding to tubulin^13^,^14^. Because the three nucleotide-binding motifs (the P-loop, Switch 1 and Switch 2) do not belong to the same subdomain, the nucleotide environment gets remodeled along with the kinesin mechanochemical cycle. The P-loop is embedded in the so-called “P-loop subdomain” that comprises elements of the N-terminal and of the C-terminal parts of the motor domain. The C-terminal part of Switch 1, together with the first residue of Switch 2, has been ascribed to the “Switch 1/2 subdomain”, internal in the sequence of the motor domain, whereas most of Switch 2 is N-terminal to the α4 helix, one of the main elements of the “tubulin-binding subdomain”^13^. These recent X-ray structural studies have been conducted in parallel with electron microscopy characterization of what occurs in a motor domain as a function of its nucleotide, culminating in about 6 Å studies of kinesin bound to microtubules that were broadly consistent with the X-ray results^15^,^16^.

One of the points that remained uncertain from these studies is that microtubule binding and nucleotide release were characterized in the same structure and, therefore, it was difficult to ascertain which structural changes were due to each of the two steps of the mechanism. One way to answer this question is to study apo-kinesin in the absence of microtubules. Mutations have been identified that accelerate nucleotide release by a kinesin from several times^13^,^17^,^18^ to several hundred-fold^19^ but the structural consequences of these mutations have only been sparsely investigated. Here we characterized kinesin-1 P-loop mutations that interfere with ADP binding and determined the structure of the corresponding mutated nucleotide-free kinesins. Remarkably, these structures are mostly similar to those of ADP-kinesin or of tubulin-bound apo-kinesin; these conformations are also adopted by the parental, nucleotide-depleted, wild-type protein. Most importantly, our results enlighten the mechanism of ADP release from kinesins.

## Results and Discussion

### Mutational approach to enhance nucleotide release from kinesin-1

**Figure 1:**
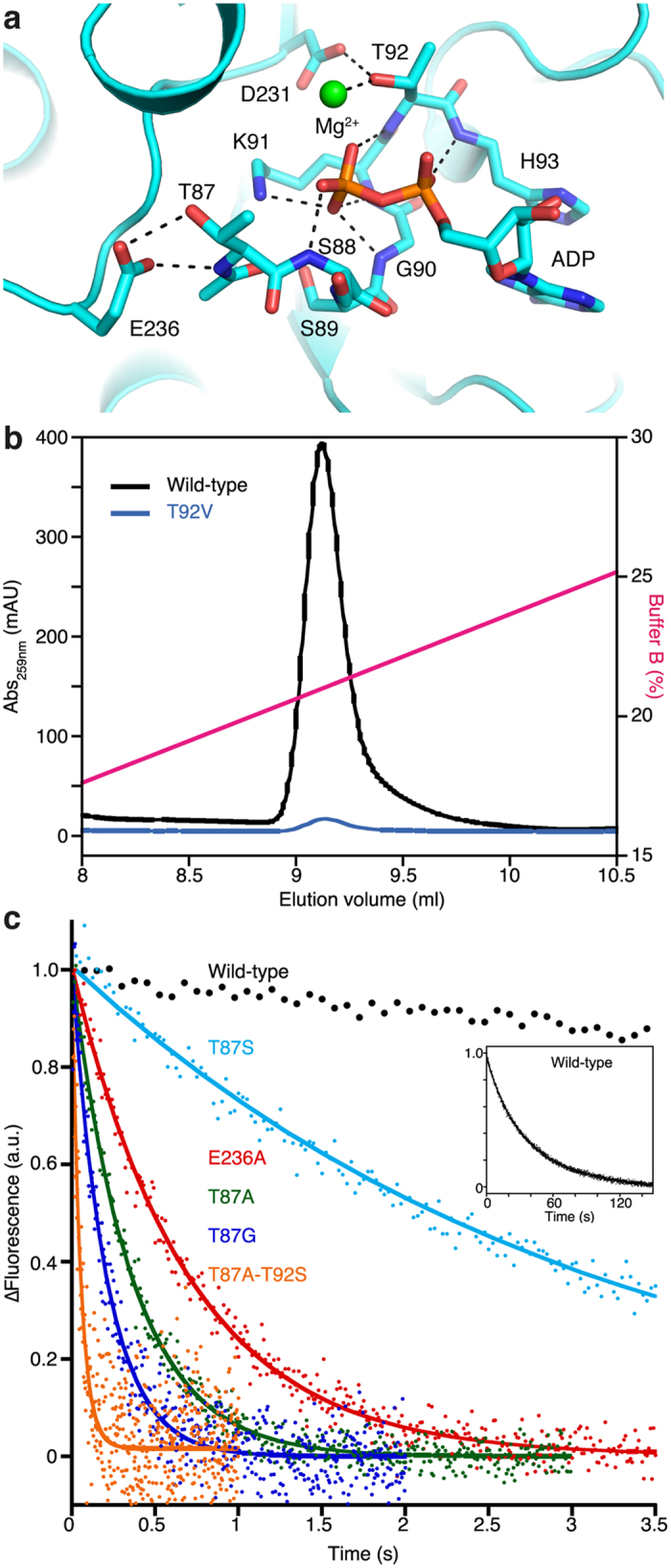
The interference of kinesin-1 mutations with ADP binding and release. (**a**) View of the ADP-kinesin nucleotide-binding site (pdb id 1BG2; ref 10). T87 and T92 P-loop residues and some of their interactions are shown. The P-loop interactions with the ADP phosphates are also shown. (**b**) The T92V mutant does not bind ADP. The nucleotide content of equivalent amounts of T92V and wild-type kinesin-1 was analyzed by ion exchange chromatography. Whereas in the case of wild-type kinesin-1 a chromatographic peak is eluted at the expected position for ADP, in the case of the T92V mutant, the signal for the peak at this position is very weak and no other peak is detected. mAU, milliabsorbance units. (**c**) Dissociation of mant-ADP from kinesin-1 or from point mutants and a double mutant, as indicated. The curves are the fit of the experimental data points with a monoexponential decay function which accounts for the fluorescence decrease corresponding to the dissociation of mant-ADP from kinesin and yields the rate constant for ADP release (Table 1). In the case of the wild-type protein, which releases ADP much more slowly, the fit (inset) includes also a linear component due to photobleaching. a.u., arbitrary units.

Mutations in two general areas of kinesin have been found to facilitate nucleotide release. The first ones are in the environment of the Mg^2+^ ion that interacts with the ADP ligand in most kinesins. Indeed, initial studies demonstrated that modulating the Mg^2+^ free concentration changes the ADP release rate in kinesin-1 (ref. 20) and in kinesin-3 (ref. 21). In kinesin-1, the only residue that interacts directly with the Mg^2+^ ion is T92 (Fig. 1a)^10^, the last residue of the P-loop motif. Either a threonine or a serine, this residue is conserved in all nucleotide-binding proteins’ P-loop motif (GxxxxGK(S/T)), which is known as the Walker A motif and required for coordinating α and β phosphates^22^. In kinesins, when a serine is found at the last P-loop position, a higher ADP release rate is observed. Such is the case of the kinesin-3 Kif14, where a high ADP release rate was deduced from the spontaneous ATPase rate of the isolated motor domain (0.88 s^−1^)^23^. As expected, when the T92S mutation was introduced in kinesin-1, ADP release was substantially accelerated, about 10-fold from 0.034 s^−1^ in wild-type kinesin to 0.33 s^−1^ in T92S (Table 1). Other mutations that interfere with the kinesin mechanism have been identified at this position in kinesin-1. It has been shown that when this residue is mutated to an isoleucine or to an asparagine, the corresponding kinesin binds to microtubules without dissociating, even in the presence of a large concentration of ATP^24^,^25^. We also found that when T92 is mutated to a valine, the nucleotide content of this mutant was very low compared with that of wild-type kinesin (Fig. 1b). Together with the observation that the T92V fluorescence does not increase upon incubation with mant-ADP, these data indicate that T92V has lost most of the kinesin affinity for ADP.

An initial indication that residues on the other side of ADP as compared to Mg^2+^ are important for nucleotide binding came from the study of the kinesin E236A mutant, which releases ADP about 100-fold faster than wild-type kinesin^19^, a result we were able to confirm (Fig. 1c, Table 1). In ADP-kinesin-1, E236 forms two hydrogen bonds with T87 in the P-loop motif, one with the main chain and the other one with the side-chain (Fig. 1a), whereas these interactions do not exist in tubulin-bound apo-kinesin^13^. Kinetic studies show that the kinesin conserved T87 residue is important for ADP binding, though it does not interact directly with the nucleotide: the T87S mutant released ADP 10 times faster than wild-type (Fig. 1c, Table 1), an effect similar to that of the equivalent mutation in *Drosophila* kinesin-1 (ref. 17) and in kinesin-14 (ref. 18). More importantly, when more mutations of residue 87 were tested, the T87A and T87G mutants were found to release ADP much faster than wild-type (k_off_ = 2.77 s^−1^ and 4.80 s^−1^, respectively) (Fig. 1c, Table 1).

To gain further understanding of the effect of nucleotide release on the kinesin, we have determined structures of the two single amino acid mutants of kinesin-1, T87A and T92V, in nucleotide-free states. The corresponding structures will now be presented.

### Apo-kinesins in an ADP-kinesin-like conformation

**Figure 2:**
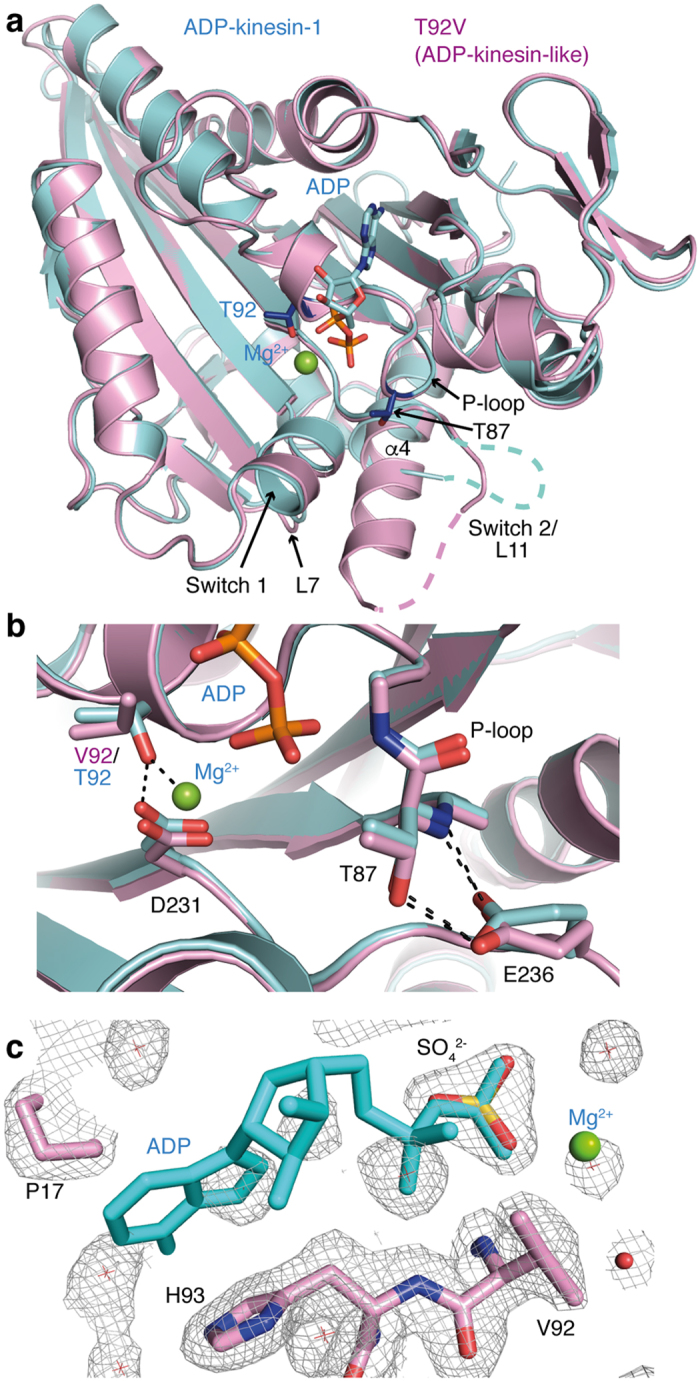
ADP-kinesin-like structure of nucleotide-free kinesin-1. (**a**) Superposition of ADP-kinesin-1 (pdb id 1BG2, blue, with the side chain of residues T87 and T92 in darker blue) and of the T92V mutant (pink, P2_1_ crystal form), indicating that both motor domains share the same overall conformation. In both ADP-kinesin-1 and T92V, a portion of Loop 11 (L11) is flexible. In T92V, several residues at the C terminal end of this loop are ordered and elongate α4. The α4 extension tilts by a few degrees to avoid a clash with Loop 7 (L7). (**b**) A close look at the T92V P-loop mutation. The valine of one of the two T92V molecules in the asymmetric unit, which is best defined (see panel c), is shown here. The T87-E236 interactions are shown both in ADP-kinesin and in T92V. (**c**) The 2 F_obs_ − F_calc_ electron density map (contoured at the 1 σ level) in the nucleotide-binding site of T92V (ADP-kinesin-like form) with a modeled sulfate ion. ADP and the Mg^2+^ ion from ADP-kinesin are shown for reference. Stereo images of the electron density maps of nucleotide-depleted wild-type kinesin-1 in the ADP-kinesin-like and tubulin-bound apo-kinesin-like forms are presented in Supplementary Fig. 2.

We first determined the 2.0 Å resolution structure of T92V in a P2_1_ space group crystal form (Supplementary Table 1). There were two similar molecules in the asymmetric unit, with a root mean square deviation (r.m.s.d.) after superposition of 0.47 Å (305 Cαs compared). The overall conformation of the protein is like that of ADP-kinesin-1 (r.m.s.d. = 0.72 Å over 300 Cαs compared to pdb id 1BG2 (ref. 10), average value for the two molecules of the T92V asymmetric unit) (Fig. 2a) and much more different from the conformation of tubulin-bound apo-kinesin-1 (r.m.s.d. = 1.37 Å over 296 Cαs compared to kinesin in the 4LNU pdb data set^13^) (Supplementary Fig. 1). Compared to ADP-kinesin-1, the three nucleotide binding motifs are conserved, as expected from the small deviation between the two structures: the Switch 1 and P-loop motifs superimpose very well, whereas a part of the L11 loop, where Switch 2 is embedded, is disordered in both structures (Fig. 2a). However, in apo-T92V, helix α4, which is C-terminal to L11, starts at residue D249 instead of residue K256 in ADP-kinesin-1, i.e. is two turns longer (Fig. 2a). The T92V α4 helix, whose elongation is likely to be stabilized by a crystal contact, is nevertheless shorter than in tubulin-bound kinesin-1, whatever the nucleotide state, where it starts at position 246 (ref. 13,14). Finally, whereas T92 interacts with D231 in wild-type ADP-kinesin-1 (Fig. 1a), this hydrogen bond is not formed in the T92V mutant, as expected (Fig. 2b).

The main difference between T92V and wild-type ADP-kinesin is the missing nucleotide and magnesium ion in the T92V nucleotide binding cleft. Instead of the nucleotide, an electron density, well accounted for by a sulfate ion that likely comes from the crystallization solution, is located close to the missing ADP β phosphate (Fig. 2c). Therefore, we conclude from these observations that the T92V mutation disables Mg^2+^ binding, which would normally stabilize the ADP association and limit its release, and leads to a nucleotide-free kinesin without the need of an overall structural change towards the tubulin-bound apo-kinesin conformation. Together with the milder effects observed with the T92S mutation (Table 1) and with the mutation to an alanine of the T92-interacting residue D231 (ref. 13), these results are consistent with a spontaneous dissociation of ADP from wild-type kinesin that is limited by that of Mg^2+^ (ref. 20).

We were also able to crystallize in the same buffer conditions the nucleotide-free parental kinesin-1 (without the T92V mutation), following nucleotide digestion with apyrase, and to determine its structure (Supplementary Table 1, Supplementary Fig. 2). Remarkably this structure is isomorphous to that of ADP-kinesin^10^. Therefore, an ADP-kinesin-like conformation can be adopted both by nucleotide-free wild-type kinesin and by the T92V mutant.

### Isolated apo-kinesins in a tubulin-bound apo-kinesin-1 conformation

Apo-T87A kinesin-1 was crystallized in a new crystal form following nucleotide digestion by apyrase and gel filtration to remove any trace of nucleotide. The crystals diffracted to 2.6 Å (Supplementary Table 1) and there were six identical molecules in the asymmetric unit (r.m.s.d. of Cαs in pairs of molecules varies between 0.3 Å and 0.55 Å). Using these T87A crystals as seeds, we were also able to crystallize wild-type apo-kinesin-1 and the T92V mutant and to determine their structures (Supplementary Table 1, Supplementary Fig. 2). Whereas most of the following analysis was performed taking the apo-T87A structure as a reference, identical conclusions could be reached from these wild-type apo-kinesin and T92V isomorphous structures.

**Figure 3:**
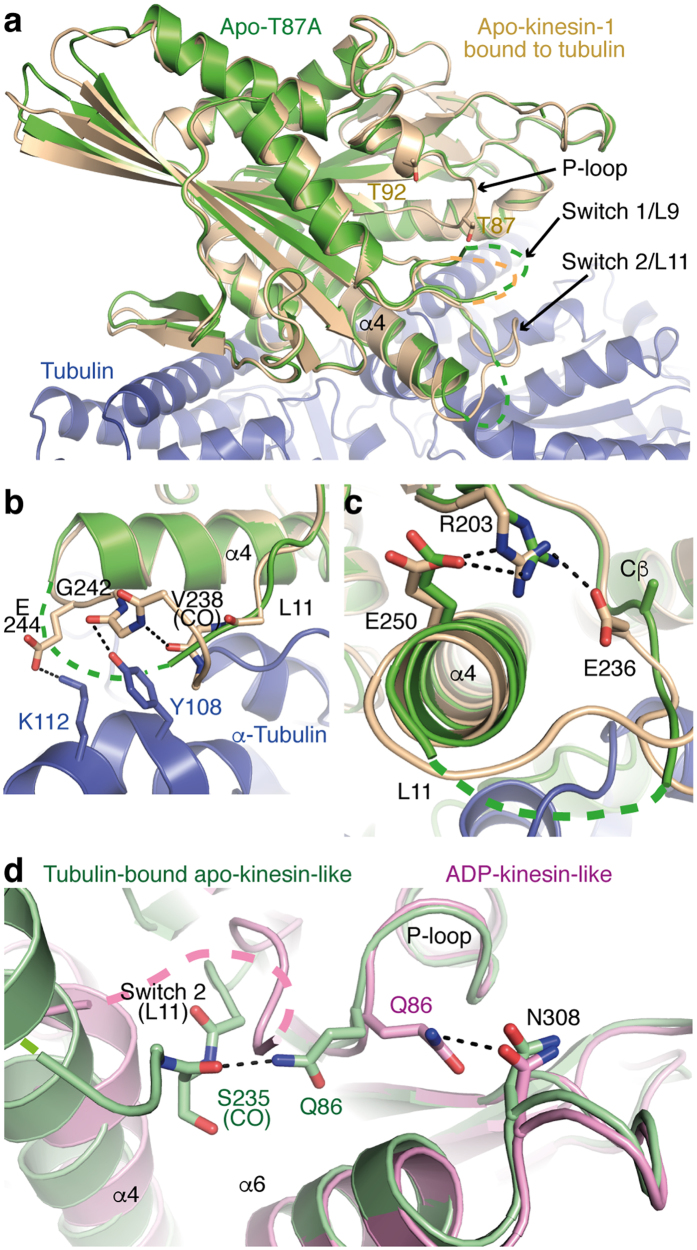
Isolated apo-kinesin-1 in a tubulin-bound apo-kinesin-1 conformation. (**a**) Apo-T87A (green) superimposed on apo-kinesin-1 (wheat, the side chain of residues T87 and T92 is drawn) in complex with tubulin (blue) (pdb id 4LNU). In both structures, L9 is disordered whereas most of L11 is ordered, leading to full elongation of the α4 helix. (**b**) The structure of the kinesin L11 loop. Whereas in the absence of tubulin, a stretch of L11 remains flexible, in tubulin-bound apo-kinesin, this stretch interacts with tubulin and L11 is fully ordered. (**c**) The side chain of residue E236 is disordered in apo-T87A. In the complex with tubulin of apo-kinesin-1, E236 is ordered and interacts with the Switch 1 residue R203. In T87A, R203 stays at a position similar to that in tubulin-bound apo-kinesin-1 and establishes a hydrogen bond with E250 in α4, but the E236 side-chain is disordered beyond Cβ. **(d)** The Q86 interaction changes in apo-kinesin-1. The structures of nucleotide-depleted wild-type kinesin-1 in the ADP-kinesin-like conformation (pink) and in the tubulin-bound apo-kinesin-like conformation (green) have been superimposed (taking the P-loop subdomain as a reference).

The r.m.s.d. of Cαs of the apo-T87A kinesin-1 compared with ADP-kinesin-1 varies between 1.2 Å and 1.5 Å. By contrast, the conformation of apo-T87A is much more similar to that of apo wild-type kinesin-1 bound to tubulin, the r.m.s.d. of Cαs being close to 0.8 Å whatever the apo-T87A molecule considered. Consistently, the secondary structure elements of both proteins superimpose very well (Fig. 3a) and very similar subdomain rearrangements are seen in both proteins, as compared with ADP-kinesin (Supplementary Fig. 1). The conformations of the three nucleotide-binding motifs are consistent with this scheme. In particular, the structure of the P-loop, which is part of the P-loop subdomain, does not change significantly between ADP-kinesin and apo-T87A or upon binding of kinesin-1 to tubulin. The structures of Switch 1 and Switch 2, the N-terminal part of the former and the C-terminal part of the latter not belonging to any of the subdomains^13^, may differ in the kinesin-1 structures that have been determined. Indeed the tip of the L9 loop (the loop to which Switch 1 belongs) is disordered both in T87A and in the initially determined apo-kinesin-1 structure^13^ whereas it folds in different conformations in the ADP-kinesin-like structure^10^ (Fig. 2a) and in tubulin-bound kinesin in an ATP-like state^14^. In contrast, apo-T87A differs from apo-kinesin-1 bound to tubulin in a small portion of the Switch 2 containing L11 loop: a stretch between Switch 2 and the α4 helix is disordered in apo-T87A whereas interactions with tubulin stabilize it in the complex (Fig. 3b).

The different stabilization of the L11 loop has consequences on the last residue of Switch 2 (E236) and on its environment. As already mentioned, in kinesin-1 in an ADP-kinesin-like conformation, E236 establishes two hydrogen bonds with T87 (Figs 1a and 2b). In apo-kinesin-1 bound to tubulin, the structure of L11 changes and E236 interacts with the Switch 1 residue R203. After ATP binding, this R203-E236 salt bridge is required for water activation and ATP hydrolysis^12^. But in apo-T87A, where the structure of L11 has changed again, the R203-E236 interaction is lost and the E236 side-chain is disordered and not visible beyond Cβ (Fig. 3c). Since it is disordered in isolated T87A apo-kinesin-1, E236 does not make any visible interaction with the P-loop and with residue 87 in particular, leading to a higher mobility of the P-loop. Indeed the temperature factors of this loop are higher than those of the kinesin central β-sheet, taken as a reference (average temperature factor of the P-loop Cαs is 47 Å^2^, over the six molecules in the asymmetric unit, to be compared to an average temperature factor of 35 Å^2^ for the central β-sheet Cαs). In contrast, the temperature factors of the P-loop are much closer to those of the central β-sheet both in ADP-kinesin^10^ and in apo-kinesin in an ADP-kinesin-like conformation (this work) (Supplementary Table 2). This last feature is also observed in the structure of an ADP-bound kinesin-14 Kar3 mutant having a Glu-to-Ala substitution at a position equivalent to that of residue 236 in kinesin-1 (ref. 26). Taken together, these observations suggest that the P-loop destabilization in nucleotide-free T87A, as well as in the isomorphous structure of wild-type apo-kinesin and of T92V (Supplementary Table 2), is the combined result of ADP release and of the loss of the T87-E236 interactions.

The structure of isolated apo-kinesin that is similar to that of apo-kinesin-1 in its complex with tubulin provides an additional opportunity to probe the changes that occur when ADP-kinesin binds to tubulin (or microtubules). In particular, residue Q86 that interacts with residue N308 in the ADP-kinesin conformation, points towards the carbonyl of S235 when the tubulin-bound apo-kinesin-like conformation is adopted (Fig. 3d). Mutating the Q86 residue to an alanine would clearly destabilize the interaction of this residue with N308 in ADP-kinesin, favoring ADP release, as witnessed by an increase of this reaction rate (from 0.034 s^−1^ to 0.14 s^−1^). This effect is further confirmed by the observation that the rate of ADP release by the double mutant Q86A-T87A is higher than that of the T87A point mutant (3.5 s^−1^ vs. 2.77 s^−1^, Table 1).

It is remarkable that mutations of T87 to alanine or glycine have among the strongest effects on ADP release measured so far, although the most obvious direct interactions of ADP with the P-loop are with the ε-amino group of K91 and with the peptidic nitrogens of residues 88 and 90 to 93 (Fig. 1a). The effect of the mutation of E236, which interacts with T87, to an alanine is also high^19^. P-loop destabilization, together with the removal of the nucleotide, leads to a tubulin-bound apo-kinesin-like conformation, as seen in the structure of the apo-T87A kinesin (Fig. 3a). In wild-type ADP-kinesin-1, this conformational change is prevented by the interaction between E236 and T87, until kinesin binds to tubulin, which displaces E236 and prevents it from interacting with T87.

### Comparison with ADP release upon microtubule binding

In a natural context, the release of ADP from kinesin upon microtubule binding proceeds in two steps, a transition from weak to strong microtubule binding followed by ADP release^6^,^27^. The corresponding structural changes lead both to the destabilization of the P-loop and of Mg^2+^ binding^13^,^15^, hence ADP release is greatly accelerated^5^. Apart from the T92V substitution, which leads to a kinesin unable to bind nucleotides (Fig. 1b), the rates of ADP release by all the mutants tested so far remain much lower than the rate of microtubule-stimulated ATP hydrolysis by kinesin-1 (k_cat_ = 50 s^−1^; ref. 14). In these mutants, two residues in the P-loop, at positions 87 and 92, were more particularly targeted, as described above. Since most of ADP is embedded in the P-loop subdomain and interacts with the P-loop, it is not surprising that interactions of that loop with the rest of the motor domain that are lost in the tubulin-bound apo-kinesin-1 conformation are important for ADP binding. As a consequence, ADP release is accelerated when they are prevented. This is in particular the case of the interactions of T87 and T92 (in the P-loop) with E236 and D231, respectively (Figs 1a and 2b). Interestingly, a substantially higher rate of ADP release was reached by the T87A-T92S kinesin double mutant (k_off_ = 19 s^−1^) compared to the single mutants (Fig. 1c, Table 1).

**Figure 4:**
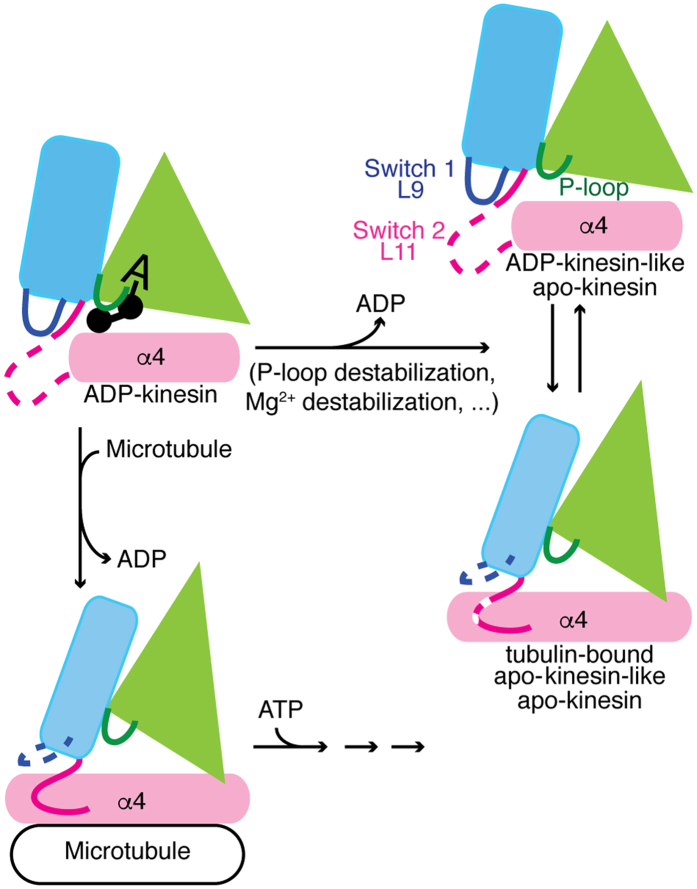
Kinesin conformational changes associated with ADP release. In the absence of microtubules, nucleotide-free kinesin may be obtained by mutating residues involved in the stabilization of the P-loop or of Mg^2+^ binding, and/or by hydrolyzing ADP. Isolated apo-kinesin alternates between the two conformations we have described, the tubulin-bound apo-kinesin-like conformation being characterized by a fully elongated α4 helix (pink), a destabilized L9 loop (blue) and a nearly fully ordered L11 loop (magenta). In a natural context, the binding of ADP-kinesin to microtubules triggers conformational changes that also lead to the destabilization of Mg^2+^ binding and of the P-loop and are associated with ADP release. In this case, the L11 loop is fully ordered. Microtubule-bound apo-kinesin is primed to bind ATP, allowing the kinesin cycle to proceed.

To sum up, our results point to two features of ADP binding, the importance of P-loop stabilization and that of the interactions with the Mg^2+^ ion. Whereas we demonstrated that, in the absence of microtubules, apo-kinesin-1 may adopt two conformations, ADP-kinesin-like and tubulin-bound apo-kinesin-like (Fig. 4), the effect of tubulin binding is to shift the balance between them and to stabilize the tubulin-bound apo-kinesin-like conformation, from which ADP is released and which is primed to bind ATP^6^ (Fig. 4). During processive movement, multiple cycles are performed, where the nucleotide gets hydrolyzed and then replaced by ATP, as required.

## Methods

### Constructs and protein purification

The constructs and mutants used in this study were obtained by standard molecular biology from a monomeric cys-light version of human kinesin-1 (ref. 19). These constructs comprise the motor domain and the neck linker (construct 1–349, used for kinetic experiments) or the motor domain catalytic core only (construct 1–325, used for crystallization). Proteins were produced in BL21 *Escherichia coli* cells grown in 2YT medium. The expression was induced with 0.3 mM isopropyl β-D-1-thiogalactopyranoside at 18 °C overnight, after the absorbance at 600 nm of the culture reached 0.7 to 0.8. Cells were harvested and suspended in buffer A (25 mM Pipes pH 6.8, 1 mM MgCl_2_, 0.5 mM EGTA, 25 μM ATP, 1 mM DTT) plus 1 mM phenylmethylsulfonyl fluoride and a protease inhibitors mix (complete EDTA free, Roche Applied Science). Two ion exchange columns were used for purification, a Hitrap SP FF column (GE Healthcare), using 20% buffer B (buffer A + 1 M NaCl) for the elution, then a Mono Q column (GE Healthcare) with a 10 to 40% buffer B gradient for elution.

To generate nucleotide-free kinesin-1 (wild-type and T87A mutant), the proteins were treated with apyrase (Sigma, 0.5 U/ml) at 4 °C overnight. For crystallization, an additional gel filtration column (Superdex 200 10/300 GL, GE Healthcare) in a buffer consisting of 16 mM Pipes pH 6.8, 0.5 mM MgCl_2_, 0.2 mM EGTA, 100 mM NaCl was performed. The protein was then concentrated, quantified using the absorbance value at 280 nm and stored in liquid nitrogen until use.

### Crystallization and structure determination

Crystallizations were performed at 293 K by vapor diffusion using the hanging drop method. T92V and nucleotide-depleted wild-type kinesin were crystallized in the ADP-kinesin-like form in a buffer consisting of 0.1 M Na-acetate pH 4.5 to 5.5, 20 to 25% (W/V) polyethylene glycol 4000, 0.2 M (NH_4_)_2_SO_4_ and 0.1 mM SrCl_2_. Apo-T87A was crystallized in a tubulin-bound apo-kinesin-like conformation in a buffer containing 50 mM Mes pH 6.5, 0.16 to 0.2 M (NH_4_)_2_SO_4_ and 30% (W/V) polyethylene glycol 5000 monomethyl ether. T92V and wild-type apo-kinesin-1 were also crystallized in these same conditions following streak seeding using T87A crystals as seeds. Crystals were harvested in the crystallization buffer supplemented with 20% glycerol, then flash-cooled in liquid nitrogen until data collection.

Data sets were collected at 100 K at the Proxima1 and Proxima 2 beam lines (Soleil Synchrotron, Saint Aubin, France) and at the ID30A1 beam line (European Synchrotron Radiation Facility, Grenoble, France). The data were processed with XDS^28^ and Aimless^29^. The structures were solved by molecular replacement with PHASER^30^ using either ADP-kinesin (pdb id 1BG2) or tubulin-bound apo-kinesin (pdb id 4LNU) as search models. The structures were iteratively refined with Buster^31^ with model building in Coot^32^. Some refinement cycles were also performed using Phenix^33^. Data collection and refinement statistics are reported in Supplementary Table 1. Figures of structural models were generated with PyMOL^34^.

### Nucleotide content analysis

T92V (60 μl at about 350 μM) and wild-type (100 μl at about 200 μM) kinesins were denatured with 1 μl trifluoroacetic acid. After removal of the denatured protein by centrifugation, the supernatant containing released nucleotide had its pH neutralized then it was loaded on a Mono Q column (GE Healthcare) equilibrated with 20 mM Tris-HCl pH 8.0. The nucleotide was eluted using an NaCl gradient. ADP and ATP solutions were injected as references to calibrate the column.

### Mant-ADP release rate measurement

As a means to evaluate the ADP release rate^20^, kinesin constructs were loaded with mant-ADP and the fluorescence decrease associated with mant-ADP dissociation from kinesin was monitored at 25 °C using a SX20 stopped-flow spectrometer (Applied Photophysics). Specifically, kinesin was first desalted using a Micro Bio-spin 6 column (BioRad) equilibrated with 25 mM Pipes pH 6.8, 2 mM MgCl_2_, 1 mM EGTA and 1 mM DTT to remove excess nucleotide. Then kinesin was incubated with a fourfold excess of mant-ADP on ice for about one hour. In the case of the mutants, because the release of ADP could be very fast, the excess mant-ADP was not removed and 0.5 μM kinesin was mixed with an equal volume of a 1 mM ADP-containing buffer in the stopped-flow cell, resulting in an ADP:mant-ADP ratio of 500:1. The fluorescence intensity (excitation at 355 nm) was recorded using a 400 nm long-pass filter. The experimental data were fitted to an exponential decay to yield the dissociation rate constant k_off_.

## Additional Information

**Accession codes:** Coordinates and structure factors have been deposited with the Protein Data Bank with accession numbers 5LT0 (ADP-kinesin-like wild-type kinesin-1), 5LT1 (ADP-kinesin-like T92V), 5LT2 (tubulin-bound apo-kinesin-like wild-type kinesin-1), 5LT3 (T87A), and 5LT4 (tubulin-bound apo-kinesin-like T92V).

**How to cite this article**: Cao, L. *et al*. The structural switch of nucleotide-free kinesin. *Sci. Rep.* **7**, 42558; doi: 10.1038/srep42558 (2017).

**Publisher's note:** Springer Nature remains neutral with regard to jurisdictional claims in published maps and institutional affiliations.

## Supplementary Material

Supplementary Information

## Figures and Tables

**Table 1:** Mant-ADP release rate from kinesins, estimated by fluorescence spectroscopy.

Construct	Wild-type	Q86A	T87S	T87A	T87G	Q86A T87A	T92S	T87A T92S	E236A
**k**_**off**_ **(s**^**−1**^)	0.034 ± 0.001	0.14 ± 0.001	0.32 ± 0.002	2.77 ± 0.037	4.8 ± 0.13	3.5 ± 0.065	0.33 ± 0. 01	19.0 ± 0.97	1.43 ± 0.017

Kinetic parameters are given as value ± s.e. (calculated from the fit)
