# Supplementary material for: The structural switch of nucleotide-free kinesin
Source: Sci Rep. 2017 Feb 14;7:42558. doi: 10.1038/srep42558 (PMC5307337; doi:10.1038/srep42558)
Supplement: Supplementary Information [file srep42558-s1.pdf]

The structural switch of nucleotide-free kinesin

Luyan Cao[1,3], Soraya Cantos-Fernandes[1] & Benoît Gigant[1,2]

[1]Institute for Integrative Biology of the Cell (I2BC), CEA, CNRS, Université Paris-Sud, Université Paris-Saclay, 91198 Gif-sur-Yvette, France

[2]Correspondence should be addressed to B.G. (benoit.gigant@i2bc.paris-saclay.fr)

[3]Present address: Institut Jacques Monod, CNRS, Université Paris Diderot, Université Sorbonne Paris Cité, 75205 Paris, France

Supporting Information

Supplementary Figures 1 and 2

Supplementary Tables 1 and 2.

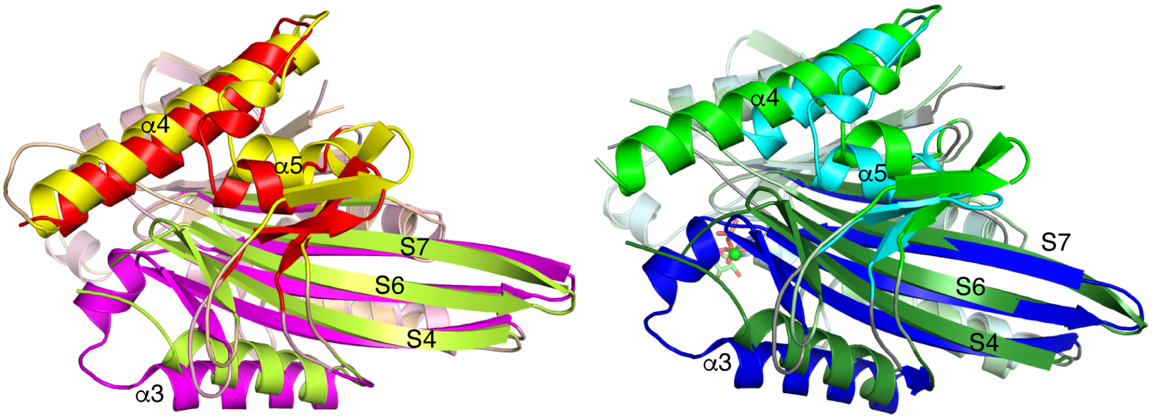

Supplementary Figure 1. The two conformations of apo-kinesin-1 studied here. (Left) Comparison of T92V (ADP-kinesin-like, $P2_1$ crystal form) with tubulin-bound apo-kinesin-1 (pdb id 4LNU). The P-loop subdomains of both kinesins have been superimposed and are shown in the background. Color code: T92V: P-loop subdomain, light pink; Switch 1/2 subdomain, magenta; tubulin-binding subdomain, red. Tubulin-bound apo-kinesin-1: P-loop subdomain, wheat; Switch 1/2 subdomain, olive; tubulin-binding subdomain, yellow. (Right) Comparison of apo-T87A with ADP-kinesin-1 (pdb id 1BG2). The P-loop subdomains of T87A and ADP-kinesin-1 have been superimposed and are shown in the background. Color code: apo-T87A: P-loop subdomain, pale green; Switch 1/2 subdomain, forest green; tubulin-binding subdomain, bright green. ADP-kinesin-1: P-loop subdomain, pale cyan; Switch 1/2 subdomain, dark blue; tubulin-binding subdomain, cyan.

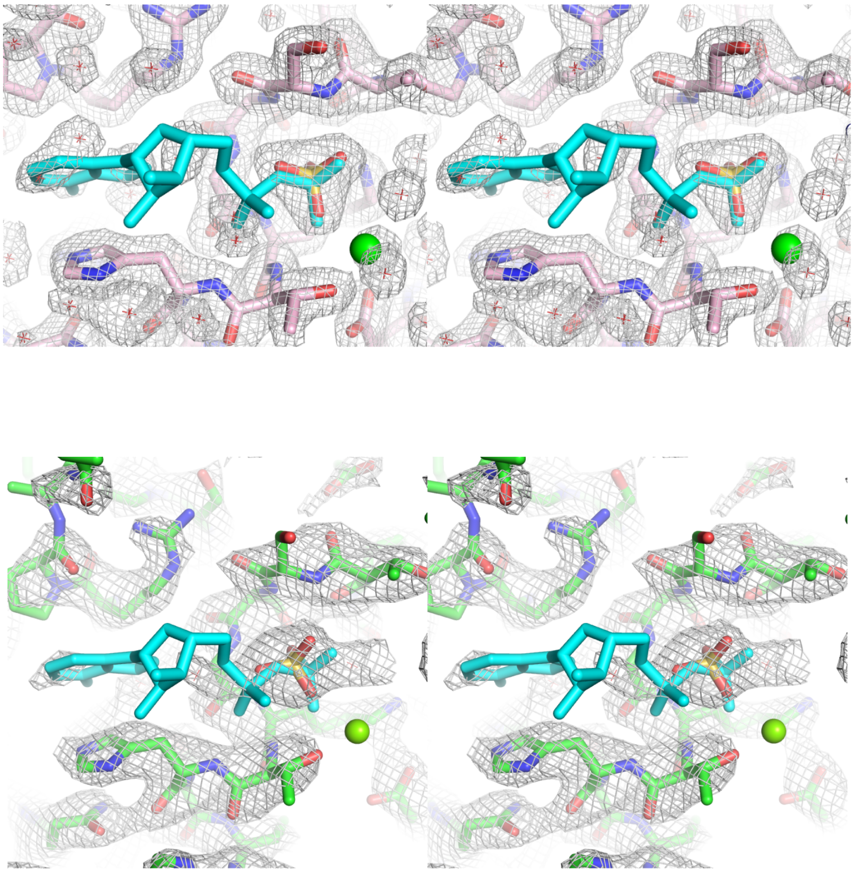

Supplementary Figure 2. Structures of nucleotide-depleted wild-type kinesin-1. (Top) Stereo view of the 2.0 Å resolution 2 F_{obs}-F_{calc} electron density map of nucleotide-depleted kinesin-1 in the ADP-kinesin-like ($P2_12_12_1$) crystal form, contoured at the 1 σ level. ADP (cyan) and the Mg^{2+} ion (green) from ADP-kinesin-1 (pdb id 1BG2) are shown for reference, after superposition of the motor domain of the two kinesins. (Bottom) Stereo view of the 2.6 Å resolution 2 F_{obs}-F_{calc} electron density map of the nucleotide-depleted kinesin-1 in the tubulin-bound apo-kinesin-like (P1) crystal form, contoured at the 1 σ level. ADP and Mg^{2+} from ADP-kinesin-1 are shown for reference, after superposition of the P-loop subdomains of the two kinesins.

Supplementary Table 1. Data collection and refinement statistics for apo-kinesin structures.

	ADP-kinesin-like form		Tubulin-bound apo-kinesin-like form		
	T92V	Wild-type	T87A	Wild-type	T92V
**Data collection**[(a)]					
Space group	$P2_1$	$P2_12_12_1$	P1	P1	P1
Cell dimensions					
a, b, c (Å)	53.9, 69.9, 100.1	49.0, 68.1, 112.4	56.7, 101.3, 101.5	56.7, 101.5, 101.6	57.2, 102.1, 102.1
α, β, γ (°)	90.0, 100.8, 90.0	90.0, 90.0, 90.0	119.6, 91.9, 91.8	119.2, 92.0, 92.0	119.5, 92.0, 92.0
Resolution (Å)	44-1.95 (2.00-1.95)	45.0-2.00 (2.05-2.00)	48.9-2.59 (2.66-2.59)	49.0-2.60 (2.67-2.60)	49.3-2.87 (2.98-2.87)
R_{meas}	0.18 (1.18)	0.17 (1.38)	0.29 (1.15)	0.23 (1.61)	0.32 (1.68)
$I / \sigma I$	6.5 (1.2)	9.2 (2.0)	3.8 (1.3)	6.8 (1.9)	4.5 (1.2)
$CC_{1/2}$	99.2 (46.0)	99.4 (67.1)	96.7 (49.0)	98.5 (60.3)	96.4 (30.8)
Completeness (%)	99.6 (96.6)	99.9 (99.9)	97.3 (91.0)	98.0 (97.6)	96.5 (80.1)
Multiplicity	3.7 (3.1)	6.4 (6.5)	2.9 (2.6)	3.5 (3.6)	3.4 (3.3)
**Refinement**					
Resolution (Å)	28.5-1.95	44.9-2.0	33.2-2.59	38.4-2.60	49.28-2.88
No. reflections	53365	26059	59376	59440	43981
Rwork / Rfree	0.195 / 0.236	0.174/0.215	0.203/0.263	0.193/0.249	0.205/0.280
No. atoms					
Protein	4813	2356	13771	13840	13833
Ligand/ion	32	18	40	55	60
Water	645	207	200	319	252
B factors					
Protein	28.0	35.3	47.7	55.1	54.6
Ligand/ion	52.8	52.4	63.1	73.2	67.3
Waters	42.3	47.1	37.7	43.7	45.4
Coordinate error (Å)	0.23	0.22	0.39	0.37	0.46
R.m.s.d.					
Bond lengths (Å)	0.008	0.007	0.009	0.010	0.011
Bond angles (°)	1.18	0.86	1.37	1.39	1.48
Ramachandran (%)					
Favored region	99.0	98.99	95.61	95.47	94.4
Allowed region	0.98	0.67	3.39	3.31	4.27
Outliers	0	0.34	1.0	1.21	1.33

[(a)]Data were collected on a single crystal. Values in parentheses are for the highest-resolution shell.

Supplementary Table 2. Average temperature factors of the Cαs of the P-loop and of the central β sheet in different structures.

Structure	Tubulin-bound apo-kinesin like [a] T87A	T92V	wild-type	ADP-kinesin like T92V[b]	wild-type	ADP-kinesin (pdb id 1BG2)
P-loop [c]	47 $Å^2$	58 $Å^2$	60 $Å^2$	16 $Å^2$	20 $Å^2$	15 $Å^2$
β sheet [d]	35 $Å^2$	43 $Å^2$	43 $Å^2$	19 $Å^2$	25 $Å^2$	21 $Å^2$
Overall (all Cαs)	47 $Å^2$	56 $Å^2$	56 $Å^2$	24 $Å^2$	30 $Å^2$	29 $Å^2$

[a] Averaged over the 6 molecules of the asymmetric unit.
[b] Averaged over the 2 molecules of the asymmetric unit.
[c] Residues 84 to 93.
[d] Residues 9 to 15, 50 to 52, 79 to 84, 126 to 136, 141 to 144, 206 to 216, 222 to 231 and 295 to 302.
